# Antibacterial Properties of Nano-Ag Coating on Healing Abutment: An In Vitro and Clinical Study

**DOI:** 10.3390/antibiotics9060347

**Published:** 2020-06-19

**Authors:** Tetsurou Odatsu, Shinichiro Kuroshima, Mika Sato, Kazuma Takase, Alireza Valanezhad, Mariko Naito, Takashi Sawase

**Affiliations:** 1 Department of Applied Prosthodontics, Institute of Biomedical Sciences, Nagasaki University, 1-7-1, Sakamoto, Nagasaki 852-8588, Japan; kuroshima@nagasaki-u.ac.jp (S.K.); sawase@nagasaki-u.ac.jp (T.S.); 2 Division of Dental Hygiene, Nagasaki University Hospital, 1-7-1, Sakamoto, Nagasaki 852-8588, Japan; m0520@nagasaki-u.ac.jp; 3 Department of Prosthetic Dentistry, Institute of Biomedical Sciences, Nagasaki University, 1-7-1, Sakamoto, Nagasaki 852-8588, Japan; ktakase@nagasaki-u.ac.jp; 4 Department of Dental and Biomaterials Science, Institute of Biomedical Sciences, Nagasaki University, 1-7-1, Sakamoto, Nagasaki 852-8588, Japan; vala@nagasaki-u.ac.jp; 5 Department of Microbiology and Oral Infection, Graduate School of Biomedical Sciences, Nagasaki University, 1-7-1, Sakamoto, Nagasaki 852-8588, Japan; mnaito@nagasaki-u.ac.jp

**Keywords:** peri-implantitis, nanosilver, antibacterial, microwave synthesis

## Abstract

Peri-implantitis is an inflammatory disease with a relevant focus on the long-term success of dental implants and implant-supported prostheses. The present study focuses on the antibacterial effect of the silver nanoparticle and investigated the suppression of dental plaque adhesion on implant abutment and/or superstructure by micro-wave assistant nanosilver coating in vivo and in vitro. Nanosilver coating on pure titanium was prepared by microwave-assisted synthesis, and characterized by scanning electron microscopy and energy-dispersive X-ray spectroscopy. In vitro studies were conducted to analyze biocompatibility using MTS assay and fluorescence microscopy with human gingival fibroblasts to evaluate antibacterial activity. During the in vivo study, nanosilver coating was applied to the healing abutments, and the prevention of plaque accumulation on nanosilver coating was confirmed by a split-mouth randomized clinical trial. The aggregation of nano-sized particles was found on the titanium surface with an antibacterial effect. The coating had no cytotoxic effect on human gingival fibroblasts. The result of the clinical trial showed that the coating suppressed the dental plaque adhesion on the healing abutments. Nanosilver coating is a promising material with antibacterial properties and can be used for implant abutments and prostheses for preventing peri-implantitis.

## 1. Introduction

To guarantee the long-term success of dental implants and implant-supported prostheses, it is important to focus on the biological complications associated with them. The consensus report of the Sixth European Workshop on Periodontology described peri-implant mucositis in approximately 80% of the subjects restored with implants, and peri-implantitis in 28–56% of the subjects [1]. The microbial composition of peri-implant infection sites showed higher counts of periodontal pathogenic bacterial species (e.g., *Porphyromonas gingivalis*, *Treponema denticola*, and *Tannerella forsythia*) compared to the healthy site [2]. *Staphylococcus aureus*, which is not part of the typical periodontal pathogen, also shows higher counts compared to the healthy site [3]. During bacterial colonization, *Staphylococcus aureus* acts as an early colonizer, creating a favorable environment for the adhesion and colonization of other bacterial species [4,5]. Therefore, *Staphylococcus aureus* seems to play an important role in the establishment and development on peri-implantitis.

To prevent supra- and sub-gingival biofilm formation and plaque accumulation, allowing antimicrobial function on implant abutment and/or prostheses would be advantageous for the prevention of peri-implantitis, which would subsequently increase the long-term success of the implant. Additionally, the healing abutments are tightened to the implant, and passing through the oral cavity predisposes it to peri-implant infections. While post-operative plaque control has been identified as an important factor in improving the outcome of periodontal surgery [6], the maintenance of plaque control in the post-operative period is complicated by pain, swelling, and risk of tooth-brushing trauma. The antibacterial coating on healing abutment would also be advantageous for preventing peri-implantitis.

The modification of titanium surfaces with antibiotics, antimicrobial peptides, and coatings of chemically-synthesized nanoparticles (e.g., Ag, Cu, Zn) have proven to be effective approaches to obtaining bactericidal effects [7,8,9,10]. Among these candidates, the use of silver-based antimicrobials has been of interest because of their wide spectrum of antimicrobial activity and low toxicity [11]. The antibacterial activity of silver ions was identified in the 19th century, being used for the management of wounds until antibiotics became the standard treatment for bacterial infections [12]. Nowadays, silver-based antimicrobials have several applications in diverse areas such as electronics, cosmetics, clothing, packaging, and medical biotechnology.

Previous studies have reported methods for the synthesis of Ag nanoparticles and preparing Ag-impregnated surfaces via the reduction of metal salts in solution [13], plasma immersion ion implantation [14,15], formation of aggregates by heating, or sputter coating with physical/chemical vapor deposition [16]. Microwave-assisted synthesis [17,18,19] is a kind of heating method for making aggregates that is characterized by a faster heating rate, less energy requirement, and a more eco-friendly nature compared to other conventional methods. The implant abutments and prostheses are usually custom-made for patients by dental technicians. It would be beneficial if the process were easy, fast, and not requiring large-scaled devices for clinical use. Thus, microwave-assisted synthesis was adopted in the present study.

Clinically speaking, antibacterial coatings for dental implant and prostheses should have at least two important characteristics: effectiveness in reducing bacteria and the ability to do so without disturbing biocompatibility. This study aimed at investigating these two properties of titanium with nano-Ag coating by microwave irradiation in vitro. Furthermore, the prevention of plaque accumulation of nano-Ag coating was confirmed by a split-mouth randomized clinical trial.

## 2. Results

### 2.1. Scanning Electron Microscopy and Energy-Dispersive X-ray Spectroscopy

**Figure 1:**
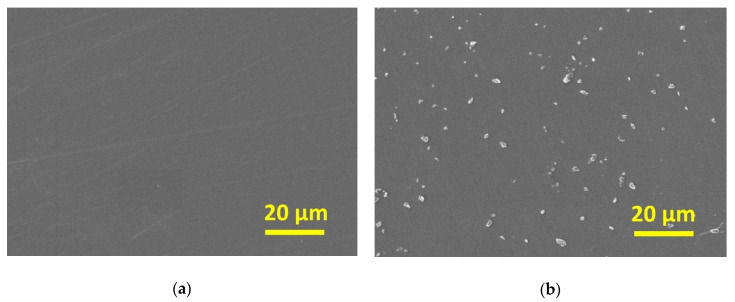
SEM images of low magnification (× 1000): (**a**) control; (**b**) nano-Ag coating. The small particles covered the nano-Ag coating.

**Figure 2:**
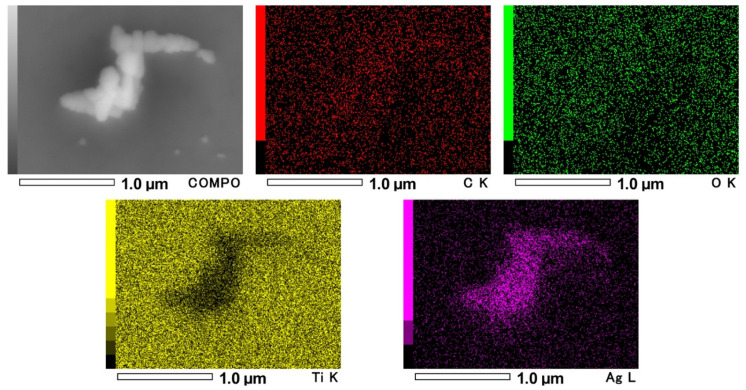
Energy-dispersive X-ray spectroscopy (EDS) mapping images of nano-Ag coating. COMPO: reflection electron composition image; C: carbon; O: oxygen; Ti: titanium; Ag: silver. The aggregation of the nano-sized particle and the composition was Ag.

The scanning electron microscope (SEM) image of control shows the remaining scratches on the surface at low magnification (× 1000) (Figure 1a). On the other hand, SEM image of nano-Ag coating shows the small particle coverage on the surface (Figure 1b). The SEM image of high magnification shows the aggregation of nano-sized particles, and the energy-dispersive X-ray spectroscopy (EDS) result reveals the composition of aggregations with Ag on the titanium disk (Figure 2). 

### 2.2. Initial Cell Attachment and Cell Proliferation

**Figure 3:**
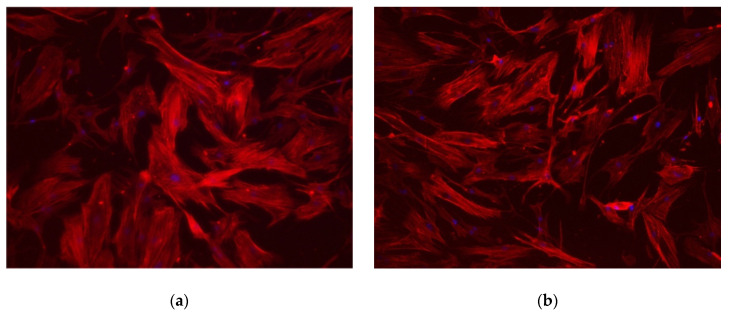
Fluorescent images after 24 h incubation: (**a**) control; (**b**) nano-Ag coating. The morphology of actin filaments (red) and nuclei (blue) indicate no difference between control and nano-Ag coating.

**Figure 4:**
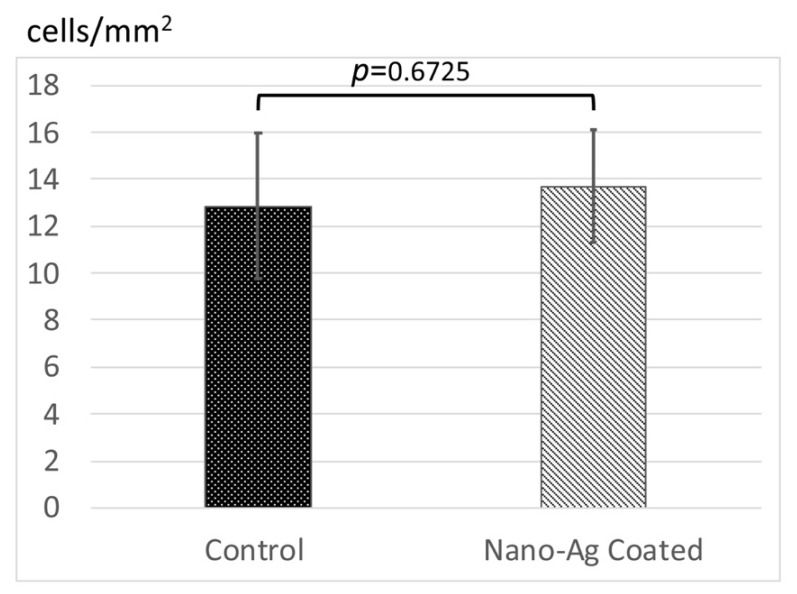
The number of cells on the samples after 24 h of incubation. Error bars represent mean ± standard deviation for *n* = 5. The one-way analysis of variance and *t*-test comparisons were used for parametric analyses. There was no statistically significant difference between control and nano-Ag coating.

Red-stained actin filaments and blue-stained nuclei on control and nano-Ag-coated titanium disks of human gingival fibroblasts (hGFs) were observed in representative fluorescent images (Figure 3). The number and shape of the cells on nano-Ag-coated titanium disks appeared to be similar to those on the control titanium disks. Indeed, the number of cells on nano-Ag-coated titanium disks was almost the same as with those on control disks (13.7 ± 2.4 cells/mm^2^ vs. 12.8 ± 3.12 cells/mm^2^, *p* = 0.6725) (Figure 4).

**Figure 5:**
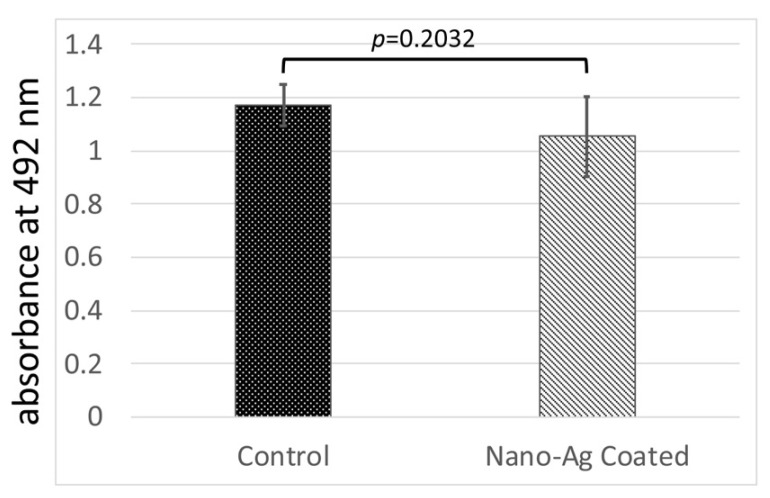
The cell proliferation assay of well plate after 72 h of incubation without medium change. Error bars represent mean ± standard deviation for *n* = 5. The one-way analysis of variance and *t*-test comparisons were used for parametric analyses. There was no statistically significant difference between control and nano-Ag coating.

Next, the proliferation of hGFs was examined using MTS assay to investigate the effect of nano-Ag coating on the biocompatibility with hGFs (Figure 5). Nano-Ag coating did not change the proliferation of hGFs when compared with that on control disks (1.169 ± 0.0785 vs. 1.053 ± 0.1485, *p* = 0.2032).

### 2.3. Antimicrobial

**Figure 6:**
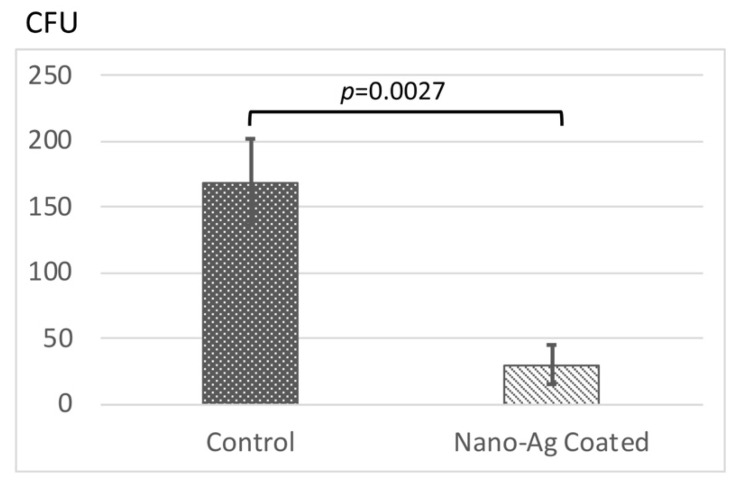
Antibacterial test according to ISO 22196: 2007(E). Error bars represent mean ± standard deviation for *n* = 3. The one-way analysis of variance and *t*-test comparisons were used for parametric analyses. Nano-Ag-coated surfaces significantly suppressed the colony-forming units (CFUs) compared to the control.

To investigate the antimicrobial effect of nano-Ag coating on *Staphylococcus aureus*, we measured the colony-forming units (CFUs) (Figure 6). The CFUs on nano-Ag-coated titanium disks were significantly suppressed when compared to those on control titanium disks (168.3 ± 32.9 vs. 30 ± 15.1, *p* = 0.0027), which indicates that nano-Ag coating provided antimicrobial activity to the titanium surfaces.

### 2.4. Clinical Study

**Figure 7:**
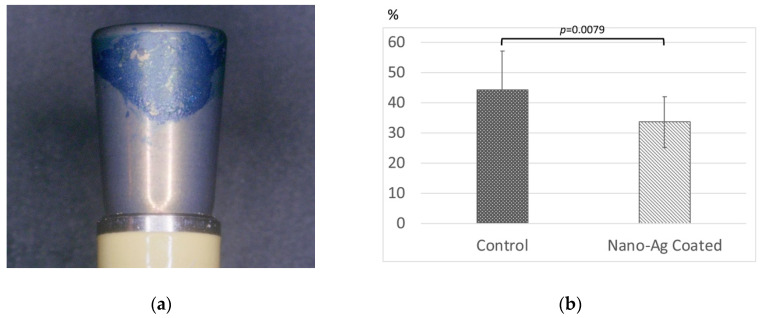
The plaque-covered area of healing abutment: (**a**) the image after staining with methylene blue disclosing solution; (**b**) the percentage of plaque-stained area to the axial surface of implant abutment. Error bars represent mean ± standard deviation for *n* = 17. One-way analysis of variance and *t*-test comparisons were used for parametric analyses. There was a statistically significant difference between control and nano-Ag coating.

The plaque-stained areas of every abutment were observed, regardless of control or nano-Ag coating (Figure 7a). There was variability of the plaque-stained area, which ranged from 20.2% to 56.8% for the nano-Ag coating and 16.2–66.6% for the control. Notably, the plaque-stained areas on nano-Ag coating were decreased in 15/17 cases. The plaque-stained area of the axial surface was significantly suppressed for the nano-Ag coating compared to the control (33.6% ± 8.35 vs. 44.3% ± 12.67, *p* = 0.0079) (Figure 7b).

## 3. Discussion

A number of treatment options have been explored for peri-implantitis. However, decontamination of the infected implant surface was only achieved by implantoplasty. Chemical agents (e.g., saline, chlorhexidine, citric acid, 35% phosphoric acid) failed to remove any biological debris, and mechanical debridements (e.g., airborne-particle abrasion, laser, titanium brush) could only remove a part of the biological debris [20]. Persson et al. tested a two-part test implant in an animal study, and re-osseointegration was achieved at the pristine site of the two-part test implant after bacterial contamination [21]. These results suggest that the quality of the titanium surface is important for re-osseointegration and it is difficult to regain the initial condition of the implant surface after bacterial contamination. Therefore, the prevention of peri-implantitis is important for the long-term success of the implants and implant prostheses.

Silver has a wide spectrum of activity against aerobic and anaerobic Gram-positive and Gram-negative bacterial strains. The mechanism of its antimicrobial activity is still not completely understood, but the consensus is that silver cations can act on a variety of targets, including interactions with bacterial cell membranes, binding to and inhibiting thiol-containing proteins, and releasing reactive oxygen species [12,22]. In this study, nano-Ag coating showed significant antibacterial activity, which is in agreement with previous studies [23,24]. hGFs are the major components of connective tissue, and they are involved in the homeostasis of collagen fibers around implant and implant abutment. Moon et al. reported that the connective tissue within 40 µm from the implant contained approximately 32% of the hGFs of all components, and they concluded that hGFs play an important role in the maintenance of a proper seal between the soft tissue and the implant [25]. Huang et al. reported that the cytocompatibility of hGF was not affected by modifying the surface with less than 10.6 at.% Ag, and typical cell cytoskeleton was maintained [23]. In this study, the initial attachment number of hGFs on the nano-Ag-coated sample was the same as on the non-coated pure titanium, and the fluorescent staining of the cell cytoskeleton suggests the cell morphology was similar both in the control and nano-Ag-coated sample. The proliferation of hGFs after 72 h of incubation without any medium change also occurred to the same extent both in the control and the test group. These results suggested that nano-Ag coating and the silver cations gradually released from the coating were not cytotoxic and had no effect on the hGFs in these experimental conditions. Kitagawa et al. reported that the positive patch test reactions of silver were exhibited in 0.7% of patients with suspected metal allergy in Japan [26]. Compared to titanium, which is considered a biocompatible metal with a low incidence of metal allergy, exhibiting 5.2% of positive reactions, we can say that the nano-Ag coating used in this study is applicable in clinical use.

In terms of the clinical trial in this study, we performed a quantitative analysis of plaque accumulation on the healing abutments, as they exhibited the same tendency as implant abutments and prostheses, and could be collected without trauma. In a recent study, healing abutments were also used to determine the influence of surface roughness on biofilm formation [27] and the composition of bacterial flora [28]. During the test period, the participants were allowed to self-clean around the healing abutments, and could use interim dentures if they needed to. In this study, the split-mouth design was adopted, and almost all subjects were set side by side. The biases from the participants of plaque control skills and inserted location were minimized. Moreover, the analytical range was limited for the axial surface of the healing abutment, biases occurred from de-laminating the nano-Ag coating through food contact, and friction of the interim dentures could be eliminated if possible. As bacterial colonization started within 30 min of implant placement [29], and was completed within 2 weeks [28,30], the test period of 28 days (± 7 days) was enough time to analyze the initial plaque accumulation. However, the repeated abutment disconnection effects on peri-implant marginal bone level [31], once the superstructures are provided, meant that they were not easily removed. Therefore, more long-term observation needs to be done, and the durability of the coating needs to be confirmed in future studies.

## 4. Materials and Methods

### 4.1. Specimen Preparation

Grade 4 pure titanium disks (Kobe Steel Ltd., Kobe, Hyougo, Japan), each with a thickness of 2 mm and a diameter of 10 mm, were used. The disks were mirror polished with silicon point (Silicone Point M2, Shofu Inc., Kyoto, Japan) and rouge (Selebright, Selec Co., Ltd., Osaka, Japan) to simulate the implant abutment surface. The disks were then ultrasonically washed with acetone, ethanol, and distilled water for 15 min each.

Nano-Ag coating was applied with microwave-assisted synthesis (Pikkasshu, Pikkasshu Ltd., Kumamoto, Japan), as per the manufacturer’s instructions. Briefly, disks were soaked in aqueous solution containing Ag ions, and then Ag ions were sticked by means of microwave for 90 s. The surface of the nano-Ag coating was observed with SEM (JCM-6000Plus, JOEL Ltd., Tokyo, Japan) and EDS (JED-2300, JEOL Ltd., Tokyo, Japan)

### 4.2. Initial Cell Attachment and Cell Proliferation

The hGFs, purchased from American Type Culture Collection (ATCC, Manassas, VA, USA), were cultured in Dulbecco’s modified Eagle’s medium (DMEM) containing 10% fetal bovine serum (FBS; Sigma-Aldrich, St. Louis, MO, USA), 1% glutamine, and antibiotics (1% penicillin/streptomycin, Sigma-Aldrich), and were incubated at 37 °C in a humidified atmosphere of 95% air and 5% CO_2_. Samples were sterilized with ethylene oxide and set in the 24-well plate. One milliliter aliquots of hGF were seeded at a density of 5000 cells/cm^2^.

After a 24-h incubation period, hGFs on the samples were stained with phalloidin (Acti-stain 555 Fluorescent Phalloidin: Cytoskeleton Inc., Denver, CO, USA) followed by fixation with acetone for 10 min at room temperature, and then mounted with the VECTASHIELD Antifade Mounting Medium with DAPI (H-1200; Vector Laboratories, Burlingame, CA, USA). The stained cells on the samples were photomicrographed and quantitatively analyzed with a fluorescence microscope (BZ-9000, KYENCE Co, Osaka, Japan) and ImageJ software (version 1.47; National Institutes of the Health, Bethesda, MD, USA).

After 72 h of incubation without medium change, hGFs in each well were assayed with MTS (CellTiter 96, Promega, Madison, WI, USA) for cell proliferation. All treatments were exchanged with the cell proliferation assay treatment (DMEM, 10% FBS, 1% glutamine, and antibiotics (1% penicillin/streptomycin), Sigma-Aldrich), 317 µg/mL MTS reagent according to the manufacturer’s protocol) and were incubated for an additional 3 h. The color of the formazan product released by the cells was analyzed using a spectrophotometer with a 492 nm filter (Multiskan FC, ThermoFisher Scientific, Waltham, MA, USA).

### 4.3. Antimicrobial Test

The Gram-positive control strain *Staphylococcus aureus* (NBRC 14462) was subcultured twice by the single colony isolation technique on Mannitol Salt Agar (Nissui Pharmaceutical Co., Ltd. Tokyo, Japan) overnight at 37 °C. The bacteria were cultured overnight at 37 °C in brain heart infusion broth (BHI; BD, Becton, Dickinson and Company, Franklin Lakes, NJ, USA), and were diluted to a concentration of approximately 1 × 10^6^ CFU/mL.

The antimicrobial activity was tested in accordance with the ISO 22196: 2007(E) [32]. Fifty microliters (0.05 mL/cm^2^) of the bacterium suspension was pipetted on each sample surface and subsequently covered with a glass cover-slip (Thomas Scientific, Swedesboro, NJ, USA) to prevent the leaking out of the suspension at the edges. After 8 h incubation at 37 °C, samples with glass cover-slips were placed in 10 mL of BHI broth, and the bacterial cells were detached from the surfaces by vigorous vortex mixing for 5 min followed by an ultrasonic disruption for 3 min. The bacterial suspensions were serially diluted in BHI broth for colony counting by using Compact Dry (Nissui Pharmaceutical Co., Ltd., Tokyo, Japan), and incubated overnight at 37 °C, and the total count of CFUs per plate for the countable dilutions was recorded. Incubation was carried out in total darkness to prevent the photocatalytic effect of the titanium surface.

### 4.4. Clinical Study

This clinical study was conducted in accordance with the declaration of Helsinki of 1975 and its subsequent amendments in 2013. The study protocol was submitted to and approved by the Clinical Research Review Board at the Nagasaki University (CRB18-0017-1). This study was registered in the Japan Register of Clinical Trials (jRCT) on 4 April 2019 (jRCTs072190002). All subjects gave their informed consent for inclusion before they participated in the study.

Nineteen patients who underwent multiple implant placement with the second stage approach were recruited at the oral and maxillofacial implant center at the Nagasaki University Hospital from April 2019 to April 2020. The inclusion criteria were as follows: (i) being aged between 20 and 85 years, and (ii) having undergone multiple implant placement with second stage approach. Exclusion criteria were: (i) a history of allergy to silver, (ii) a history of delayed healing after implant insertion, (iii) a plan of soft tissue augmentation at the second surgery, (iv) the implant was applied to a reconstruct site, and (v) the individual being a participant of any other clinical study involving the oral cavity. A total of 17 participants (6 men and 11 women; mean age of 64.6) successfully completed the study.

At the second surgery, distal 2 implants were enrolled for this study. One of the healing abutments was treated with nano-Ag coating (“Nano-Ag Coated”), and the other was not treated (“Control”). Prior to the surgery, professional mechanical tooth cleaning was carried out by the dental hygienist. Following the administration of a local anesthetic, a full-thickness flap was reflected. The cover screws were removed, and the healing abutments were randomly fixed to each implant. Randomization was carried out with the permuted block method. Flaps were closed with 5-0 sutures. Antibiotics (amoxicillin) and analgesics (Non-Steroidal Anti-Inflammatory Drugs) were prescribed post-operatively. Then, 7 to 14 days after the second surgery, sutures were removed. Fourteen days (± 7 days) after the second surgery, the equipment for the preparation of provisional restorations was taken. Twenty-eight days (± 7 days) after the second surgery, the provisional restorations were set and the healing abutments were collected for observation.

The evaluation of the plaque coverage on the healing abutment surface was conducted as follows [33]. First, the abutment was rinsed in distilled water and then stained with methylene blue disclosing solution (0.25% m/v in distilled water, KATAYAMA CHEMICAL Ltd., Osaka, Japan) for 1 min, and then rinsed with distilled water. The healing abutments were mounted on the implant replica and the axial surface images were taken from 4 directions using a digital microscope (VHX-5000, KEYENCE Co., Osaka, Japan). The captured images were analyzed using photo-editing software (Adobe Photoshop CS4 Extended, Adobe System Co., Ltd., San Jose, CA, USA). The total axial surface area and stained area as evaluated by the number of pixels was measured, and the percentage surface covered by the plaque was calculated.

### 4.5. Data Analysis and Statistics

Quantitative data were presented as the mean ± standard deviation. To determine distribution, we examined the data rows using Levene’s test. One-way analysis of variance and *t*-test comparisons were used for parametric analyses. Values of *p* < 0.05 were considered statistically significant.

## 5. Conclusions

The in vitro and clinical studies showed the antimicrobial effects of nano-Ag coating by microwave-assisted synthesis. The clinical study section confirmed the prevention of plaque accumulation by the nano-Ag coating. These data suggest that nano-Ag coating is a promising material with antibacterial properties and can be used for implant abutments and prostheses for preventing peri-implantitis.
